# Regulation and Functions of the *lms* Homeobox Gene during Development of Embryonic Lateral Transverse Muscles and Direct Flight Muscles in *Drosophila*

**DOI:** 10.1371/journal.pone.0014323

**Published:** 2010-12-15

**Authors:** Dominik Müller, Teresa Jagla, Ludivine Mihaila Bodart, Nina Jährling, Hans-Ulrich Dodt, Krzysztof Jagla, Manfred Frasch

**Affiliations:** 1 Division of Developmental Biology, Department of Biology, University of Erlangen-Nuremberg, Erlangen, Germany; 2 Brookdale Department of Molecular, Cell and Developmental Biology, Mount Sinai School of Medicine, New York, New York, United States of America; 3 Genetics, Reproduction and Development, Institut National de la Santé et de la Recherche Médicale U931, Centre National de la Recherche Scientifique UMR6247, Clermont University, Clermont-Ferrand, France; 4 Department of Bioelectronics, Institute of Solid State Electronics (FKE), Vienna University of Technology, Vienna, Austria; 5 Section Bioelectronics, Center for Brain Research, Medical University of Vienna, Vienna, Austria; 6 Department of Neurobiology, University of Oldenburg, Oldenburg, Germany; Katholieke Universiteit Leuven, Belgium

## Abstract

**Background:**

Patterning and differentiation of developing musculatures require elaborate networks of transcriptional regulation. In *Drosophila*, significant progress has been made into identifying the regulators of muscle development and defining their interactive networks. One major family of transcription factors involved in these processes consists of homeodomain proteins. In flies, several members of this family serve as muscle identity genes to specify the fates of individual muscles, or groups thereof, during embryonic and/or adult muscle development. Herein, we report on the expression and function of a new *Drosophila* homeobox gene during both embryonic and adult muscle development.

**Methodology/Principal Findings:**

The newly described homeobox gene, termed *lateral muscles scarcer* (*lms*), which has yet uncharacterized orthologs in other invertebrates and primitive chordates but not in vertebrates, is expressed exclusively in subsets of developing muscle tissues. In embryos, *lms* is expressed specifically in the four lateral transverse (LT) muscles and their founder cells in each hemisegment, whereas in larval wing imaginal discs, it is expressed in myoblasts that develop into direct flight muscles (DFMs), which are important for proper wing positioning. We have analyzed the regulatory inputs of various other muscle identity genes with overlapping or complementary expression patterns towards the cell type specific regulation of *lms* expression. Further we demonstrate that *lms* null mutants exhibit reduced numbers of embryonic LT muscles, and null mutant adults feature held-out-wing phenotypes. We provide a detailed description of the pattern and morphology of the direct flight muscles in the wild type and *lms* mutant flies by using the recently-developed ultramicroscopy and show that, in the mutants, all DFMs are present and present normal morphologies.

**Conclusions/Significance:**

We have identified the homeobox gene *lms* as a new muscle identity gene and show that it interacts with various previously-characterized muscle identity genes to regulate normal formation of embryonic lateral transverse muscles. In addition, the direct flight muscles in the adults require *lms* for reliably exerting their functions in controlling wing postures.

## Introduction

The musculatures in both vertebrate and invertebrate animals are composed of a large variety of different muscles that are distinguished according to their specific size, morphology, and physiological properties. Whereas much progress has been made in defining the regulatory processes of myogenesis as such, the understanding of the developmental mechanisms underpinning muscle diversity is much less complete. To date, the fruit fly *Drosophila* has been one of the most profitable models for dissecting the mechanisms regulating muscle diversity. In this system, a number of mechanisms that provide specific identities to individual muscles have been described, particularly for the development of larval muscles during embryogenesis. By contrast, our knowledge about the diversification of adult muscles, which takes place in a second round of muscle development during metamorphosis, is still much more limited.

Larval muscle development in *Drosophila* leads to the formation of ∼30 distinct muscle fibers arranged in a stereotyped pattern within each embryonic trunk hemisegment (reviewed in [1]). A large body of evidence has revealed that each of these muscles is seeded by a single myoblast, termed muscle founder cell, which already retains a defined identity that predetermines the characteristics of the particular muscle it will form. Upon myoblast fusion between founder myoblasts and fusion-competent myoblasts, which largely lack distinct identities, the identity of the founder cell is then imposed on the growing muscle syncytium and shapes its development. In the current view, the identity of individual founder cells is conferred through the expression and function of particular combinations of muscle identity genes (reviewed in [2], [3]). The muscle identity genes that have been characterized functionally to date all encode various types of transcription factors. The best-characterized muscle identity factors belong to the families of the homeodomain proteins (including Apterous/Ap, Slouch/S59, Ladybird/Lb, Even-skipped/Eve), Zinc-finger factors (Krüppel/Kr), basic helix-loop-helix factors (Nautilus/Nau), and the COE (Col/Olf1/EBF) transcription factors (Collier/Col) [4], [5], [6], [7], [8], [9], [10], [11]. In addition, the activities of these identity factors are modulated by transcription factors that are expressed within distinct broad domains in the somatic mesoderm, such as the homeodomain proteins Tinman/Tin, Muscle segment homeodomain/Msh, Six4, and Pox meso [12], [13], [14], [15]. Another notable example of this latter class of regulators are the Hox factors, which are expressed in broad domains along the anterior-posterior axis within the somatic mesoderm and are known to modulate the activities of muscle identity factors in a region-specific manner along this axis [16], [17], [18]. As has been shown in some of these cases, different muscle identity genes and regional regulators are part of hierarchical and cross-regulatory networks during the development of a particular muscle. As a result, some of the identity factors and regional factors are expressed only transiently whereas the expression of others continues until a mature fiber is formed. Ultimately, the functions of these transcription factors in muscle fate determination must be mediated by their transcriptional target genes, but our information about these targets and their roles in making muscles distinct is currently rather limited [19], [20].

During metamorphosis, the majority of the adult muscles are generated anew from the descendants of undifferentiated myoblasts, termed adult muscle precursors, that are set aside during embryogenesis and start proliferating during larval stages [21] (reviewed in [22], [23]). Currently, there is no clear evidence for muscle identity genes acting at the level of individual muscle founder cells during adult muscle development. However, like in embryos, Hox genes are known to play an important role during the regional diversification of muscle patterns along the anterior-posterior axis [24], [25], [26]. Another example for genes involved in adult muscle diversification is *ladybird,* which is widely expressed in leg disc-associated myoblasts and required for normal leg muscle development [27], [28]. Hence, this embryonic muscle identity gene is redeployed during metamorphosis to participate in the control of the development of large subset of myoblasts, namely those forming the leg muscles. An instructive example of myoblast diversification during metamorphosis has also been described in the wing disc. The wing disc-associated myoblasts generate two fundamentally different types of muscles, which on the one hand include the indirect flight muscles that power the flight, and on the other hand the direct flight muscles that control wing positioning during steering and flight stabilization. It has been demonstrated that the myoblasts giving rise to the indirect flight muscles (IFMs), which form the majority of the wing disc-associated myoblasts and are located in proximal areas of the wing disc of the presumptive notum, are marked by the expression of the homeobox gene *vestigial*/*vg*. Conversely, the myoblasts forming the direct flight muscles (DFMs), which are located in adjacent areas near the future wing hinge, are marked by high-level expression of the homeobox gene *cut*. In this latter population of myoblasts, high levels of Cut repress *vestigial*, whereas in the IFM-forming population of myoblasts Vg down-regulates *cut* to low expression levels. In addition, Vg represses *apterous* (*ap*), which can therefore only be activated in the high-*cut* myoblasts. *ap* then helps specifying these myoblasts as DFM myoblasts [29], [30], [31], [32]. Altogether, these regulatory interactions and the functions ascribed to *vg* and *cut*/*ap* in IFM versus DFM development point to some mechanistic analogies of muscle diversification during larval and adult muscle development.

As the currently-known collection of muscle identity genes is still not sufficient to explain the entire muscle pattern during embryogenesis, and even less so during formation of adult muscle diversity, our laboratories have been aiming to identify additional genes of this type. In this report, we describe a new homeobox gene, which we call *lateral muscles scarcer* (*lms*), that fulfils the criteria for a muscle identity gene. During embryogenesis, *lms* is expressed specifically in the founders and syncytial fibers of the lateral muscles LT1-LT4 as part of a regulatory network that includes *ap*, which exhibits a closely related expression pattern, as well as *lb*, *Kr*, and *msh*. We show that null mutations for *lms,* which are homozygous viable, cause defects in LT muscle development that consist of a reduction in the number of muscles and morphological aberrations. These defects occur with a relatively low expressivity, similar to those reported for *ap*, and double mutants for *lms* and *ap* show additive effects. During adult muscle development, *lms* is expressed in wing disc-associated myoblasts within a small area that overlaps with the presumptive DFM myoblasts marked by high-*cut* expression. The held-out wing phenotype of *lms* null mutant flies is compatible with a requirement of *lms* for normal DFM differentiation. Because detailed analysis of the DFMs in *lms* mutant flies showed that the DFMs are present and lack any overt morphological alterations, it appears that *lms* is needed for the acquisition of the requisite functional properties of these muscles rather than their formation and morphogenesis.

## Results

### *CG13424*/*lms* orthologs are present in dipteran, chordate, and cnidarian lineages

**Figure 1:**
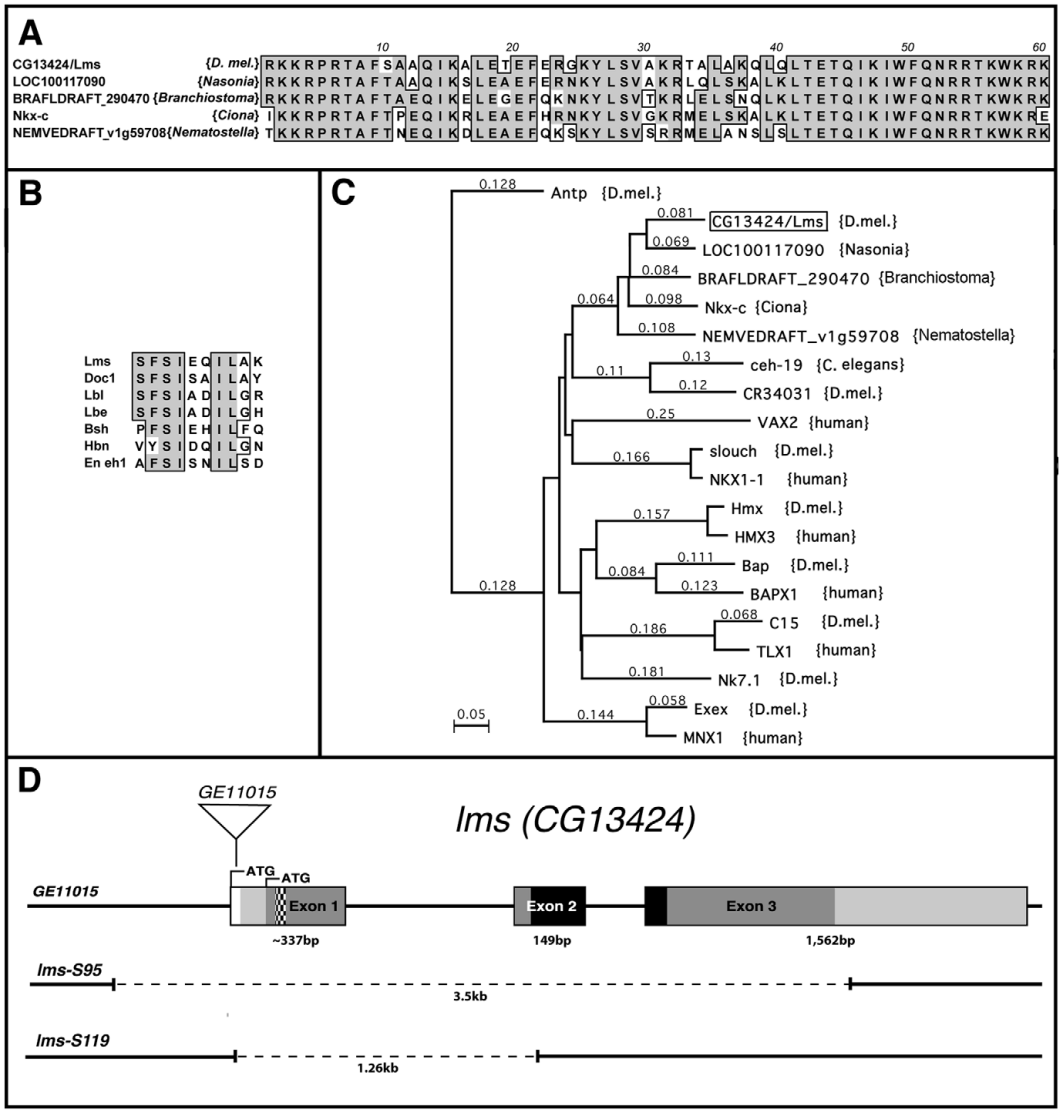
Sequence conservation of Lms and imprecise P-excisions at the *lms* locus. (**A**) Sequence alignments of the homeodomains from Lms (D. melanogaster) with its predicted orthologs from the sequenced genomes of *Nasonia vitripennis* (parasitoid wasp), *Branchiostoma floridae* (amphioxus, shown is one of two paralogs also known as Nedxa and Nedxb [60]), *Ciona intestinalis* (vase tunicate), and *Nematostella vectensis* (Cnidaria). (**B**) Sequence alignments of the most closely related eh1 (TN) domains from *Drosophila* transcription factors with that from Lms. (**C**) Phylogenetic tree using the homeodomain sequences from Lms (*D. melanogaster*) and the most closely related homeodomains from *Branchiostoma*, *Nasonia*, *Ciona*, *Nematostella*, *D. melanogaster*, and humans, showing that *Drosophila* and humans lack any paralogs and orthologs, respectively, of *lms*. (**D**) *lms* gene locus with P-insertion *GE11015* and deletions generated via imprecise excision of this P-element underneath. Predicted exons are boxed, sequences covered by the longest known EST RE33150 are shaded. The open reading frame starting from the first ATG of RE33150 is shown in dark grey, the eh1 (TN) domain checkered, and the homeodomain in black. The open reading frame extends towards the 5′ in the genomic sequence to another ATG that is embedded in a less favorable translation initiation sequence and may not be present in the transcripts.

The *Drosophila* homeobox gene *CG13424*, subsequently named *lateral muscles scarcer* (*lms*), was reported to be expressed in specific somatic muscle founders (Berkeley *Drosophila* Genome Database, [33]), which suggested it as a candidate for a new regulator of muscle identity or differentiation. Database searches showed that orthologs of *lms* are present in the parasitoid wasp *Nasonia*, honey bee, the Cnidarian *Nematostella*, as well as in the chordates *Branchiostoma* and *Ciona* (Fig. 1A and data not shown), thus indicating an ancient origin of this gene. By contrast, in vertebrates and in *C. elegans* orthologs of *lms* are not present, suggesting that the corresponding genes have been lost in these lineages. Lms also contains a putative eh1 repression domain (in NK homeodomain proteins also known as TN domain) near its N-terminus (Fig. 1B) [34]. A phylogenetic tree for *lms* and its closest homologs in the above species as well as humans is shown in Fig. 1C, which indicates a sequence affinity of the Lms homeodomain with the NK homeodomain family. The *Drosophila lms* gene locus, which maps to 57A on the second chromosome, is shown in Fig. 1D.

### Expression pattern of *lms* during embryonic development

**Figure 2:**
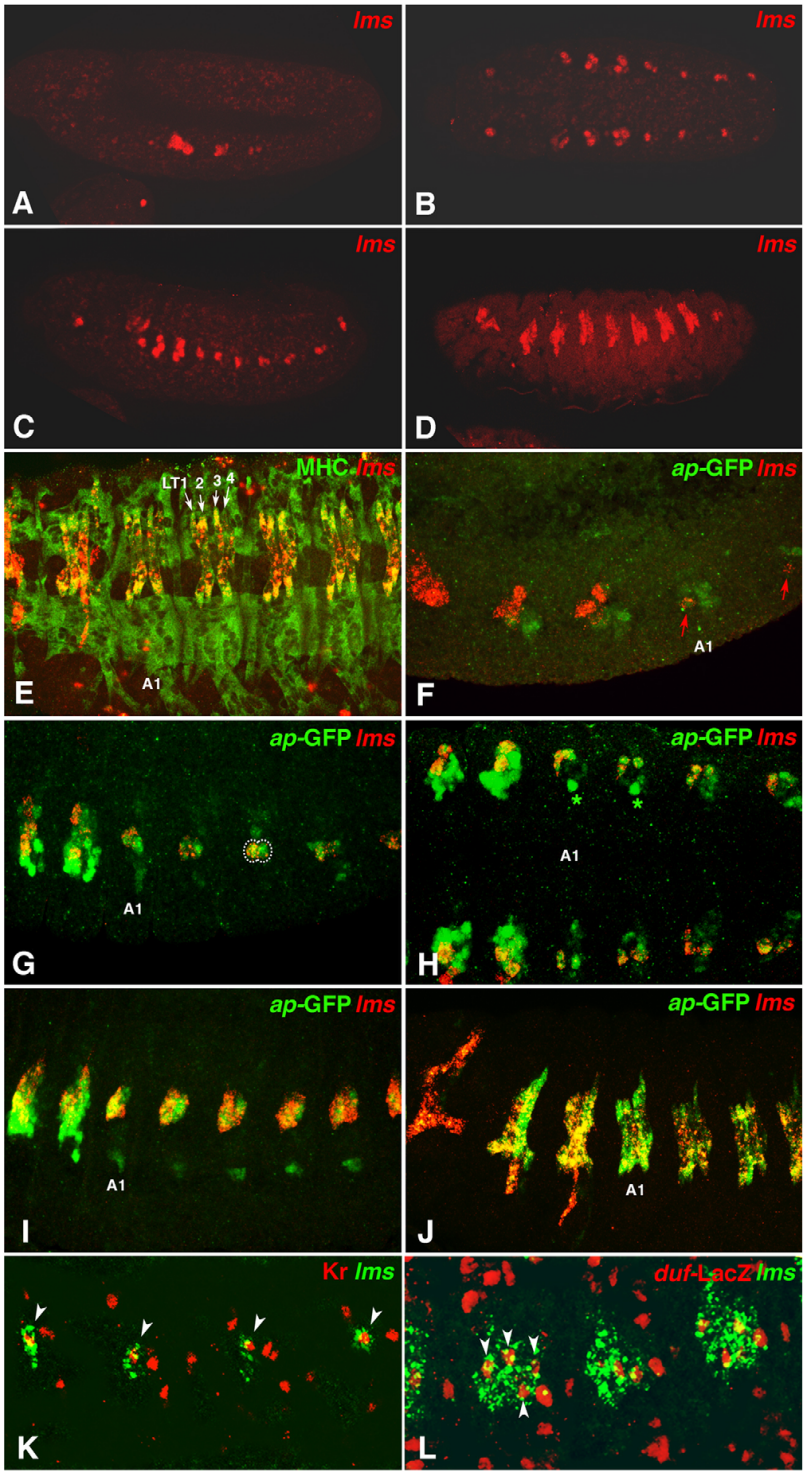
*lms* mRNA expression during embryonic lateral transverse muscle (LTM) development. (**A**) Stage 11 embryo (lateral view) showing earliest *lms* mRNA expression in thoracic clusters of somatic mesodermal cells. (**B**) Early stage 12 embryo (ventral view) showing *lms* mRNA expression in thoracic and abdominal progenitors of lateral transverse muscles (LTMs). Sites of procephalic expression may correspond to progenitors of yet unidentified head muscles (see also C, D). (**C**) Late stage 12 embryo (lateral view) showing *lms* mRNA expression in founder cells that begin to fuse to form LTMs. (**D**) Stage 15 embryo (lateral view) showing *lms* mRNA expression in LTM fibers. (**E**) High magnification view of stage 16 embryo counter-stained with MHC antibodies (green), which identifies the *lms* expressing muscles (red) of the thoracic segments T2 and T3 and the abdominal segments as LTM 1–4 (muscles 21–24) (A1: first abdominal segment). (**F**) Stage 11 *apME680-GFP* embryo showing lateral high magnification view of segments T1 to A2 stained for *lms* mRNA (red) and GFP protein (anti-GFP; green). In T2 and T3, *lms* expression partially overlaps with the *ap*-GFP expressing clusters, and in abdominal segments, *lms* expression occurs in a single cell within the *ap*-GFP clusters at this stage (arrow). (**G**) Early stage 12 *apME680-GFP* embryo (T2–A5) showing coexpression of *lms* mRNA (red) and *ap*-GFP (green) in two muscle progenitors in each abdominal segment (circled), with the anterior cell displaying higher *lms* mRNA levels. (**H**) Mid stage 12 *apME680-GFP* embryo (T2 –A4, ventral view) showing co-expressed *lms* mRNA (red) and *ap*-GFP (green) in 2–3 LTM muscle progenitors and/or founder cells per abdominal hemisegment. An additional, *lms*-negative ap-GFP-labeled cell appears ventrally adjacent that will form a ventral acute muscle (asterisks; see also I). (**I**) Stage 14 *apME680-GFP* embryo (T2 – A6, laterally) showing co-expression of *lms* mRNA (red) and *ap*-GFP (green) in LTM muscle precursors. (**J**) Stage 15 *apME680-GFP* embryo (T1 – A4, laterally) showing coincidence of *lms* mRNA (red) and *ap*-GFP (green) expression in abdominal LTM1-4 muscles, partial overlap in LT1-4 of T2 and T3, and exclusive *lms* expression in muscle VT1 of T1. (**K**) Stage 12 lateral high magnification view of embryo stained for *lms* mRNA (green) and Kr protein (red). The presumed founder cell of LT2 expresses both *lms* and Kr (arrow heads). (**L**) Late stage 12 lateral high magnification view or *rP298-lacZ* embryo stained for *lms* mRNA (green) and LacZ. Each *lms* cell cluster contains four founder nuclei for the muscles LT1 – 4 (arrow heads).

The embryonic expression pattern of *lms* was analyzed in greater detail by whole mount *in situ* hybridizations of wild type embryos and by comparing it to additional markers for the somatic mesoderm (Fig. 2). The gene lacks any maternal contribution and its expression occurs exclusively in the somatic mesoderm. During stage 11, *lms* mRNA is first detected in small clusters of mesodermal cells in each of the three thoracic hemisegments (Fig. 2A). During early stage 12, *lms* transcripts are also detected in abdominal segments, initially in a single mesodermal cell in each abdominal hemisegment (Fig. 2B). At late stage 12 and into early stage 13, the abdominal *lms* expression consists of clusters of three to four cells in each hemisegment and the size of the *lms*-positive thoracic cell clusters is increased (Fig. 2C). As shown in Fig. 2D in a stage 15 embryo, these *lms*-positive cell clusters develop into lateral somatic muscle fibers. Double stainings with a myosin heavy chain (MHC) antibody, which marks all somatic muscles, identifies the four *lms* positive syncytia in each abdominal segment as the Lateral Transverse muscles (LT1 - LT4, also known as muscles 21–24; Fig. 2E, [1]). LT1–LT4 are also positive for *lms* in the thoracic segments T2 and T3, whereas in T1, where LT muscles are absent [1], *lms* is expressed in muscle VT1 (see below, Fig. 2J).

The muscle identity gene *apterous* (*ap*), encoding a LIM homeodomain containing transcription factor, is also expressed within the four LT muscles, as well as in two Ventral Acute muscles (VA2 and VA3) [35]. To compare the temporal and spatial expression patterns of *lms* and *ap* in these muscles and their progenitors, embryos carrying the *apME680*-GFP reporter were used for GFP/*lms* mRNA double stainings. The apME680 enhancer element recapitulates mesodermal *ap* expression in LT1–LT4 and in a single Ventral Acute muscle (VA) and their corresponding progenitors [35]. *ap*-GFP expression can be observed during stage 11 in small cell clusters within abdominal segments before *lms* expression is detectable (data not shown). During early stage 12, the single *lms*-positive cell observed in each abdominal hemisegment corresponds to one of the *ap*-GFP-positive cells within each abdominal cell cluster, whereas in T2 and T3 most of the *lms*-expressing cells are located dorsally adjacent to the *ap*-GFP-expressing cells (Fig. 2F). In T1, only *lms* expression but no *ap*-GFP can be detected (Fig. 2F). During mid stage 12, a second cell becomes positive for *lms* in each abdominal hemisegment and *ap*-GFP becomes restricted to the same two cells (Fig. 2G; the original, anterior cell has accumulated higher levels of *lms* mRNA). In T1 and T2, *ap*-GFP expression expands into the *lms*-positive cells (Fig. 2G). Double stainings for *lms* and the muscle identity factor Krüppel (Kr) at stage 12, which is expressed in the muscles LT2 & LT4 and their founders [6], show that the initial *lms*-positive cell corresponds to one of the Kr-positive cells (likely the LT2 founder or LT1/LT2 progenitor; Fig. 2K).

During late stage 12, *lms* and *ap*-GFP are co-expressed in up to four cells per abdominal hemisegment, which likely correspond to the four founder cells of the LT1 - 4 muscles (Fig. 2H). This assignment agrees with the presence of four founder nuclei labeled by *duf* (*rP298*)-LacZ within each segmental cluster of *lms* expression during late stage 12 (Fig. 2L, arrow heads). Upon myoblast fusion during stage 14, *lms* and *ap*-GFP are co-expressed in the thoracic and abdominal LT muscle precursors, although in the thorax *ap*-GFP still extends further ventrally (Fig. 2I; *ap*-GFP is also seen in *lms*-negative abdominal VA muscle precursors). Finally, in the fully-formed muscle fibers of the four Lateral Transverse muscles (LT1–LT4) from T2, T3, and each abdominal hemisegment, *lms* transcripts and *apterous*-GFP remain co-expressed, whereas in T1 only *lms* expression can be detected (Fig. 2J).

Taken together, these observations strongly indicate that *lms* and *ap* are co-expressed in the four founder cells of the Lateral Transverse Muscles LT1–LT4 and in the muscle fibers formed from them, although the spatial and temporal dynamics leading to this co-expression differs for the two genes.

### Regulatory interactions among different muscle identity genes during LT muscle development

**Figure 3:**
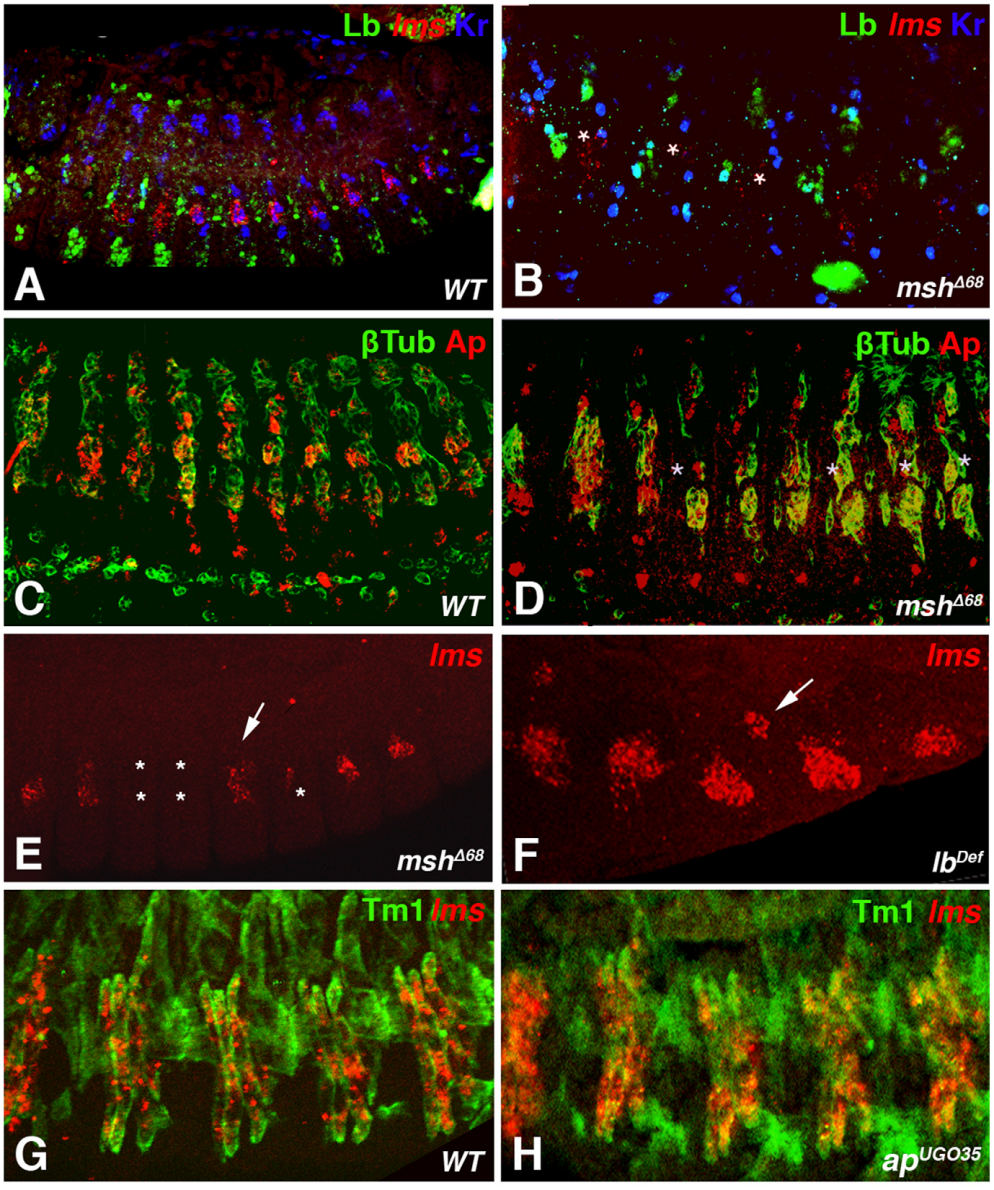
Effects of mutations of the muscle identity genes *msh*, *lb*, and *ap* on *lms* expression. (A) to (D) show lateral and (E, F) dorso-lateral views of stage 13 embryos stained to reveal the effects of *msh* and *lb* loss of function mutations on expression of *lms* or *ap*. Triple-stained embryos for anti-Lb anti-Kr and *lms* transcripts are shown in (A and B) and double-stained for β3-tubulin (βTub) and *ap* transcripts in (C and D). (**A**) and (**C**) Wild type embryos. (**B**) Strongly reduced *lms* expression in homozygous *msh^Δ68^* embryos is associated with the loss of Kr staining in LT2 and LT4 muscles and with expanded Lb expression domain (SBM muscle). (**D**) In segments with reduced *ap* expression in the absence of *msh*, β3-tubulin-labeled muscle precursors are still detected. (**E**) and (**F**) show *lms* expression patterns in homozygous *msh^Δ68^* and in *ladybird*-deficient embryos (homozygous *Df(3R)3/1*). *msh* and *lb* mutations have opposite effects on *lms* expression. In *msh* null mutants (E) *lms* transcripts are either absent in all or in a subset of LT muscle precursors (asterisks) or the *lms* expression levels are reduced (arrow). Loss of *lb* (F) leads to an expanded expression of *lms* in lateral domains. Occasionally, ectopic expression of *lms* in more dorsal clusters of muscle cells can be detected (arrow). (**G, H**) Lateral views of stage 15 embryos (G: wild type, H: homozygous *ap^UGO35^*; four abdominal hemisegments are shown with a focus on LT muscles) stained for *lms* transcripts and with anti-TM1 to reveal the muscle pattern, showing that in *ap* mutant embryos *lms* expression is largely unaffected.

Apart from *lms* and *ap*, the muscle identity gene *muscle segment homeobox* (*msh*) is active in the founder cells of muscles LT1 - 4, and *Krüppel* (*Kr*) is expressed in LT2 & LT4 and their founders (among founders of additional muscles) [6], [13]. Conversely, the *ladybird* (*lb*) genes are expressed in the progenitors and founders of the segment boundary muscles (SBM) and lateral adult muscle precursors, which arise at positions directly adjacent to those of LT1 - 4 (Fig. 3A) [6], [7], [13]. To determine whether *msh, ap,* and *lb* exert any positive or negative regulatory inputs towards *lms*, we examined the effects of their loss or gain of function on *lms* expression.

As shown in Fig. 3B (see also Table 1), deletion of *msh* results in a severe reduction of *lms* and a loss of *Kr* expression in the founders of the LT muscles. In addition, the expression of *ap* in the founders and precursors the LT muscles is strongly reduced (Fig. 3D, asterisks, compare with Fig. 3C). At later stages, when myoblast fusion has initiated, *lms* expression does appear in the precursors of the LT muscles in *msh* mutants, but the expression is still reduced or absent in many of the segments (Fig. 3E). These effects are in line with the reported loss of many LT muscles in *msh* mutants [13] and suggest that this loss may in part be due to the failure in the proper activation of these three muscle identity genes in their muscle founders.

**Table 1:** Quantification of genetic effects on embryonic *lms* expression.

*lms* expression/genetic context	Unchanged*n/%	Enlarged**n/%	Reduced**n/%	Ectopicn/%	Lossn/%	Total hemisegments
*lb ^def^*	17/36%	20/42%	0	10/22%	0	47
*24B>lbe*	12/29%	0	30/71%	0	0	42
*msh* ^Δ*68*^	9/19%	0	28/60%	0	10/21%	47

*less than 30% difference of expression area between wt and a genetic context measured on projections from confocal stacks with detection of *lms* transcripts. Age matched stage 13 embryos oriented laterally were imaged and counted (Olympus, Fluoview Analysis tool was used for calculating expression area).

**more than 30% difference of expression area (mm^2^) between wt and a genetic context measured on projections from confocal stacks with detection of *lms* transcripts. Age matched stage 13 embryos oriented laterally were imaged and counted (Olympus, Fluoview Analysis tool was used for calculating expression area).

In embryos carrying a deficiency for the two *lb* genes, *lbe* and *lbl, lms* expression in lateral clusters appears slightly expanded (Fig. 3F; Table 1). Occasionally, *lms* expression is also seen in a few ectopic mesodermal cells that lie close to the segmental borders, possibly at the positions of SBM myoblasts that have lost their identity in the *lb^def^* context.

As shown in Fig. 3H for late stage *ap^UGO35^* null mutants, loss of *ap* function does not grossly affect *lms* expression in LT muscles (and their founders, data not shown). As has been reported, mutation of *ap* causes occasional losses of individual LT muscles [5] (see also below). As we observe that any changes of *lms* expression in *ap* mutants closely parallel these effects on the numbers of LT muscles, these changes could be an indirect consequence of mis-specification of individual muscle fates rather than direct effects of *ap* on *lms* expression. The mild effects of *ap* mutation on *lms* expression could either be due to the absence of regulatory inputs or to functional redundancy of *ap* with other regulators.

**Figure 4:**
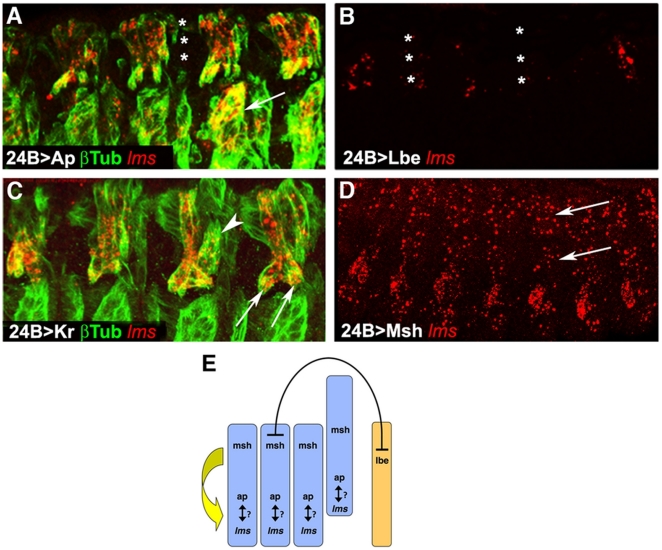
Effects of ectopic mesodermal expression of the muscle identity genes *ap*, *lbe*, *Kr*, and *msh*, on *lms* expression. (**A**) Lateral view of stage 15 *24B-GAL4;UAS-ap* embryo stained with anti-β3-tubulin (βTub) and for *lms* transcripts. Four abdominal hemisegments are shown revealing that panmesodermal Ap expression leads to an ectopic activation of *lms* in ventral muscles (arrow). Asterisks indicate position of segment border muscle (SBM) that is barely detected in the Ap gain of function context. (**B**) Lateral view of stage 14 *24B-GAL4; UAS-lbe* embryo stained for *lms* transcripts and showing four abdominal segments. In the *lbe* gain of function context *lms* expression is lost or reduced (asterisks). (**C**) Lateral view of stage 15 *24B-GAL4;UAS-Kr* embryo stained with anti-β3-tubulin (βTub) and for *lms* transcripts, showing four abdominal segments. Kr gain of function leads to muscle pattern defects such as LT muscle bending (arrows) or LT muscle shortening (arrowheads) without affecting *lms* expression. (**D**) Lateral view of stage 14 *24B-GAL4; UAS-msh* embryo stained for *lms* transcripts. *lms* expression is ectopically induced in mesodermal cells at dorso-lateral and dorsal locations (arrows). (**E**) Scheme showing regulatory interactions between *msh, lbe, ap* and *lms* identity genes in lateral muscles. LT1-LT4 are in blue whereas SBM is in yellow (all interactions are meant to be cell-autonomous and shown as they occur during the muscular precluster, progenitor, and founder cell stages). It is currently not known whether the interactions shown are direct or whether they are indirect, e.g., being a result of cell fate transformations.

To complement the loss of function studies with gain of function experiments, we forced the expression of *ap*, *lbe*, *Kr*, and *msh* pan-mesodermally by using the *24B*-*Gal4* driver in combination with the corresponding UAS-constructs [36]. Panmesodermal expression of *ap* leads to malformation of the Lateral Transverse muscles (LT1 - 4, Fig. 4A) [17]. Additionally, a loss of the SBM (♯8) muscle can be detected (Fig. 4A, asterisks). Notably, *ap* misexpression results in *lms* expression in the three Ventral Acute muscles (VA1 - VA3, Fig. 4A, arrow). In the wildtype situation, no *lms* expression can be observed in these muscles.

Ectopic expression of *lbe* via *24B-Gal4* line results in enlarged and duplicated SBMs, increased numbers of lateral adult muscle precursors, and other muscle defects [7]. With regard to *lms*, we observe a severe reduction, and in some segments complete loss, of mRNA expression within the areas of the presumptive LT muscles (Fig. 4B; Table 1). It has been shown that increased SBM formation in *ladybird* mis-expressing embryos occurs at the expense of the LT musculature, which is replaced by unfused mono-nucleated myoblasts [7]. Based on these data and on the observed effects of *ladybird* loss and gain of function on *lms*, we conclude that *ladybird* may normally repress LT muscle founder genes, including *lms*, in SBM founders, whereas ectopic expression of *ladybird* is able to repress *lms* (and additional identity genes) in neighboring LT founders, leading to their failure to form LT muscles.

Pan-mesodermal *Kr* expression leads to mis-arrangements and patterning defects of the somatic musculature, including reduced numbers and altered shapes of the LT muscles (Fig. 4C). The remaining LT muscles are often bent at their ventral endings and others appear thicker and shortened (Fig. 4C, arrows and arrow head). In these embryos, *lms* mRNA is still expressed within the residual LT muscles. The absence of any ectopic expression of *lms* suggests that *Kr* is either not sufficient to activate *lms* on its own or it lacks any inputs altogether towards *lms* regulation.

Ectopic expression of *msh* via *24B-Gal4* leads to patterning defects in the dorsal musculature as well as disorganization of the ventral muscles [13]. Mis-expression of *msh* has also an effect on the expression of *lms*. Beside the regular expression of *lms* within the LT muscles, ectopic *lms* expression in lateral and in particular in dorsal areas of the somatic mesoderm is observed (Fig. 4D, arrows).

Taken together, misexpression of *msh* and to a lesser extent of *ap* causes ectopic expression of *lms*, suggesting that in the normal situation these two muscle-identity genes contribute to the transcriptional activation and maintenance of *lms* expression in the founders and precursors of the developing LT muscles. Conversely, our data suggest that *ladybird* normally has repressive inputs on *lms* expression, analogous to its reported effects on the muscle identity gene *slouch*, in order to prevent the inappropriate activation of these genes in the progenitors and founders of SBM muscles and lateral adult muscle precursors by yet undefined upstream regulators (Fig. 4E).

### Generation of *lms* null alleles and consequences of loss of *lms* for LT muscle development

For the analysis of *lms* function during muscle development we generated null alleles by using the *GE11015* P-element insertion in the *lms* locus (Fig. 1D). *GE11015* is inserted 11 ntd. downstream of the computationally predicted start of the open reading frame, although there is currently no experimental evidence that this part of the gene locus is transcribed and that the ATG upstream of the insertion is being used as a translation start codon. The facts that the 5′ of the longest available EST begins ∼100 bp downstream of the insertion site, the second ATG of the predicted open reading frame has a better match to *Drosophila* consensus sequences for translation start sites, and the *GE11015* strain is fully viable with normal embryonic expression of *lms* (data not shown) would suggest that *GE11015* is inserted just upstream of the transcription start site of *lms*. From an imprecise excision screen with *GE11015* we recovered several semilethal lines that we characterized by genomic PCR and sequencing of the deletion breakpoints. One allele, *lms^S95^*, carries a deletion of both sides flanking the insertion site and removes the complete coding sequence of *lms* without affecting the coding sequences of the two adjacent genes located on either side. The deletion from a second allele, *lms^S119^*, extends from the insertion site into the beginning of exon 2 (Fig. 1D). Based upon these molecular defects, both alleles are predicted to be functional nulls. Due to their weakness, adult homozygous flies for neither of the two alleles can be maintained as homozygous lines.

**Figure 5:**
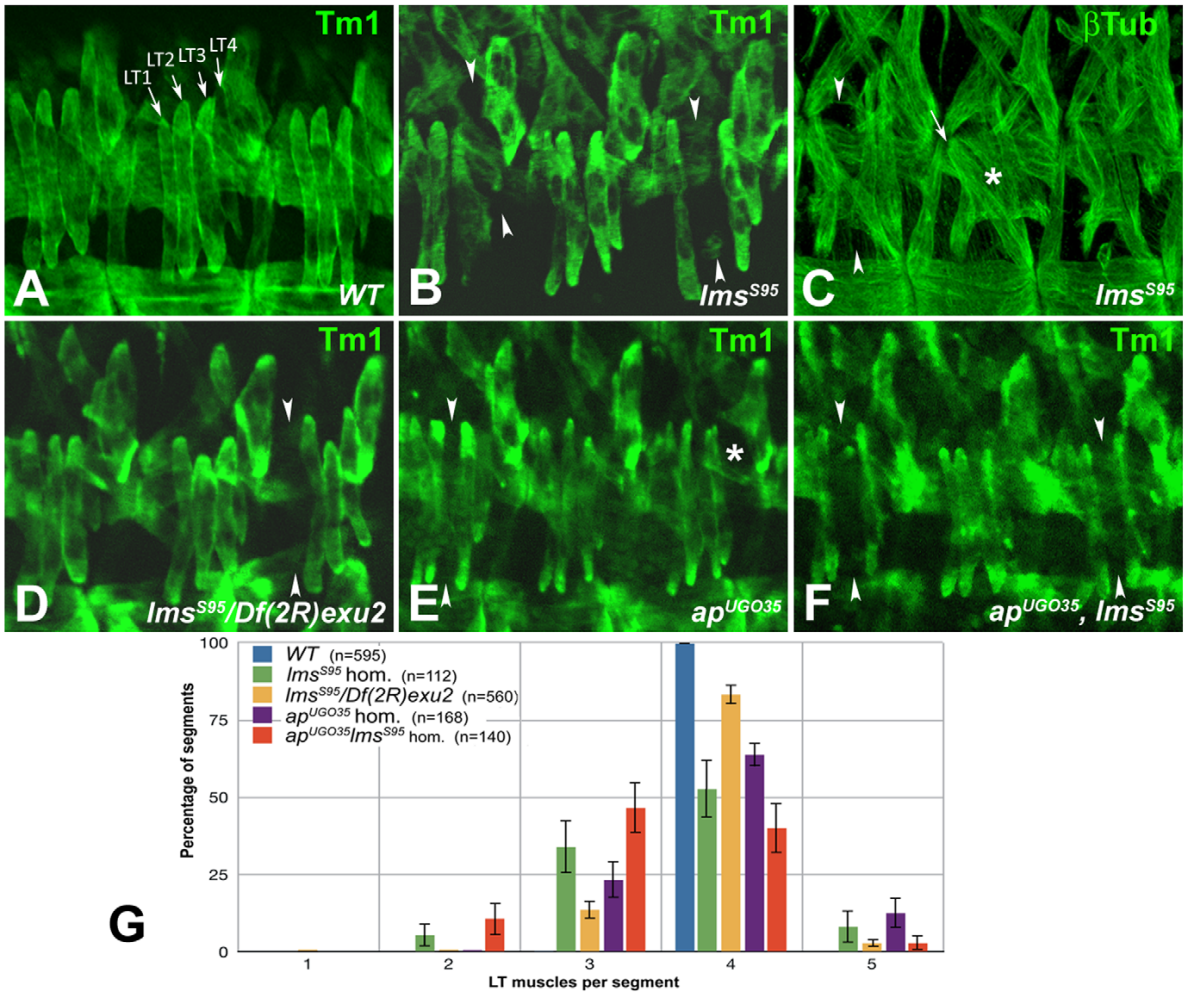
Embryonic LT muscle phenotypes in *lms* mutants and *ap*, *lms* double mutants. (A) to (F) show representative examples of phenotypes in three abdominal segments from stage 16 embryos with the denoted genotypes that were stained with anti tropomyosin (Tm1) or anti β3-tubulin (βTub) as indicated to visualize muscle patterns. (**A**) Wild type embryo with four LT muscles (arrows) in every segment. (**B**) Homozygous *lms^S95^* embryo with a LT4 and a LT3 muscle missing in the segments depicted on the left hand and right hand side, respectively (arrow heads). (Note that due to the slightly more dorsal orientation of the embryo as compared to embryo in (A), the VL muscles are not included in the Z-scan.) (**C**) *lms^S95^* with stronger than average disruptions of LT muscles. In left hand segment, one LT muscle is missing (arrow heads), and in segment in center, LT1 is connected to an abnormal attachment site (arrow) whereas the remaining LT muscles are replaced by aberrant syncytia with undefined identities (asterisk). (**D**) *lms^S95^/Df(2R)exu2* embryo with an LT1 muscles missing (arrow heads). (**E**) Homozygous *ap^UGO^* ^35^ embryo with mis-arranged LT muscles, causing a gap (arrow heads), and possible transformation of LT4 (asterisk) into a second LT3 muscle. (**F**) Homozygous *ap^UGO^* ^35^, *lms^S95^* double mutant embryo with only three LT muscles in each of the left and right hand segments (arrow heads; perhaps with the regular LT3 missing and LT4 transformed into LT3). (**G**) Numerical evaluation of LT muscle phenotypes. Genotypes are color-coded as shown, with “n” denoting the assessed number of abdominal segments for each genotype. Confidence intervals were calculated at 95% confidence levels. Note that sporadic occurrences of a fifth LT muscle have also been reported for controls and may be genetic background-dependent [7].

The embryonic muscle phenotypes of *lms* mutants were examined with general muscle markers and by crossing in *apME680-GFP* as a specific marker for the LT muscle pattern. In stage 16 embryos homozygous for *lms^S95^*, we frequently observe the loss of one of the LT muscles in individual abdominal segments (Fig. 5B, compare with A). Generally, the LT muscles that are still present exhibit normal morphologies and attachments. However, in rarer cases, especially when two LT muscle fibers are missing in a hemisegment, the remaining LT muscles can be attached to segmental instead of intrasegmental attachment sites (Fig. 5C). The same range of phenotypes is also observed in embryos homozygous for *lms^S119^* (data not shown) and in embryos with the excision alleles *in trans* to the larger deficiency, *Df(2R)exu2* (Fig. 5D). Overall, the observed muscle phenotypes are very reminiscent of the LT muscle phenotypes reported for *ap* mutant embryos [5] (Fig. 5E). Because *lms* and *ap* are co-expressed in developing LT muscles and mutations in both genes cause relatively mild LT muscle phenotypes, we generated *ap, lms* double mutant embryos to test whether the two genes exhibit partial functional redundancy in regulating LT muscle formation. However, in *ap, lms* double mutant embryos most of the LT muscles are also being formed (Fig. 5F). For a more detailed analysis of possible genetic interactions between *lms* and *ap* during LT muscle development, we undertook a quantitative analysis of the LT muscle phenotype of double mutant embryos and compared it to those of the single mutants. As shown in Fig. 5G, the expressivity of the LT muscle phenotype in *lms* single mutant embryos (green and yellow columns) is similar to that of *ap* single mutants (purple columns). In *ap, lms* double mutant embryos (red columns), the number of hemisegments with only three or two LT muscles present is significantly higher than for each of the single mutant genotypes. However, the effects appear additive rather than interactive, which indicates that the two genes function in parallel to ensure the formation of normal numbers of LT muscles.

### *lms* function during adult muscle development in *Drosophila*

**Figure 6:**
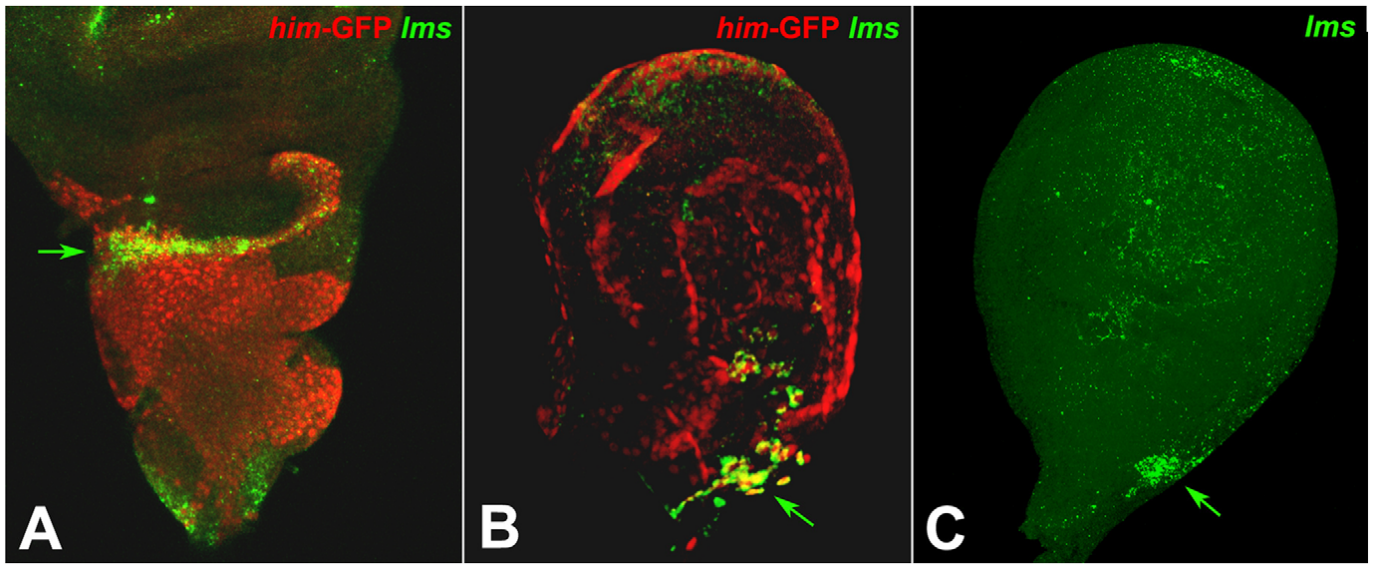
Expression of *lms* in adepithelial cells of wing and leg discs. Shown are 3rd instar wing and leg discs. (A, B) show GFP-expressing adepithelial cells associated with wing (A) and leg (B) imaginal discs dissected from the transgenic *Him-GFP* larvae and stained with anti-GFP (red) and for *lms* transcripts (green). (**A**) *lms* transcripts accumulate specifically in a subset of adepithelial cells located in most distal positions of the thoracic part of the wing disc (arrow). (**B**) In the late 3^rd^ instar leg discs from Him-GFP larvae *lms* is expressed in a restricted subpopulation of adepithelial cells (arrow) located outside of the leg disc proper within the stalk region. (**C**) Highly restricted *lms* expression in leg discs at the same position as in (B) (arrow) is already detected in early 3^rd^ instar larvae.

To examine whether *lms* also fulfils a role during the development of the adult musculature, we stained imaginal discs for *lms* mRNA expression. We used discs from a line expressing GFP driven by regulatory regions of the *Him* gene, which is expressed in all adepithelial cells of the wing and leg discs that will form the adult musculatures of the thorax and its appendices [30], [37], [38], [39], [40]. As shown in Fig. 6A, during late third instar the *Him*-GFP-labeled adepithelial cells cover the entire notum and hinge area of the wing disc. By contrast, *lms* is only expressed in the adepithelial cells within a narrow zone along the distal border of this area, which is located near the hinge region of the disc. The adepithelial cells of this zone are known to give rise to the direct flight muscles (DFMs) and, as seen in Fig. 6A, *lms* expression occurs predominantly within the anterior ∼1/2 of this zone. Likewise, *lms* is also restricted to a small area of adepithelial cells in third instar leg discs (Fig. 6B). The *lms* expressing cells are located in the stalk region of the disc outside of the leg proper area. Because clonal analysis had suggested that direct flight muscle 51 is derived from the leg disc, it is conceivable that some of this expression corresponds to myoblasts of presumptive DFM 51 in mesothoracic leg discs [38]. However, the observed expression in similar domains in all leg discs (including those of T1 and T3) would suggest that most of these cells give rise to muscles other than the DFMs, possibly including some that extend from the thorax into the coxae.

**Figure 7:**
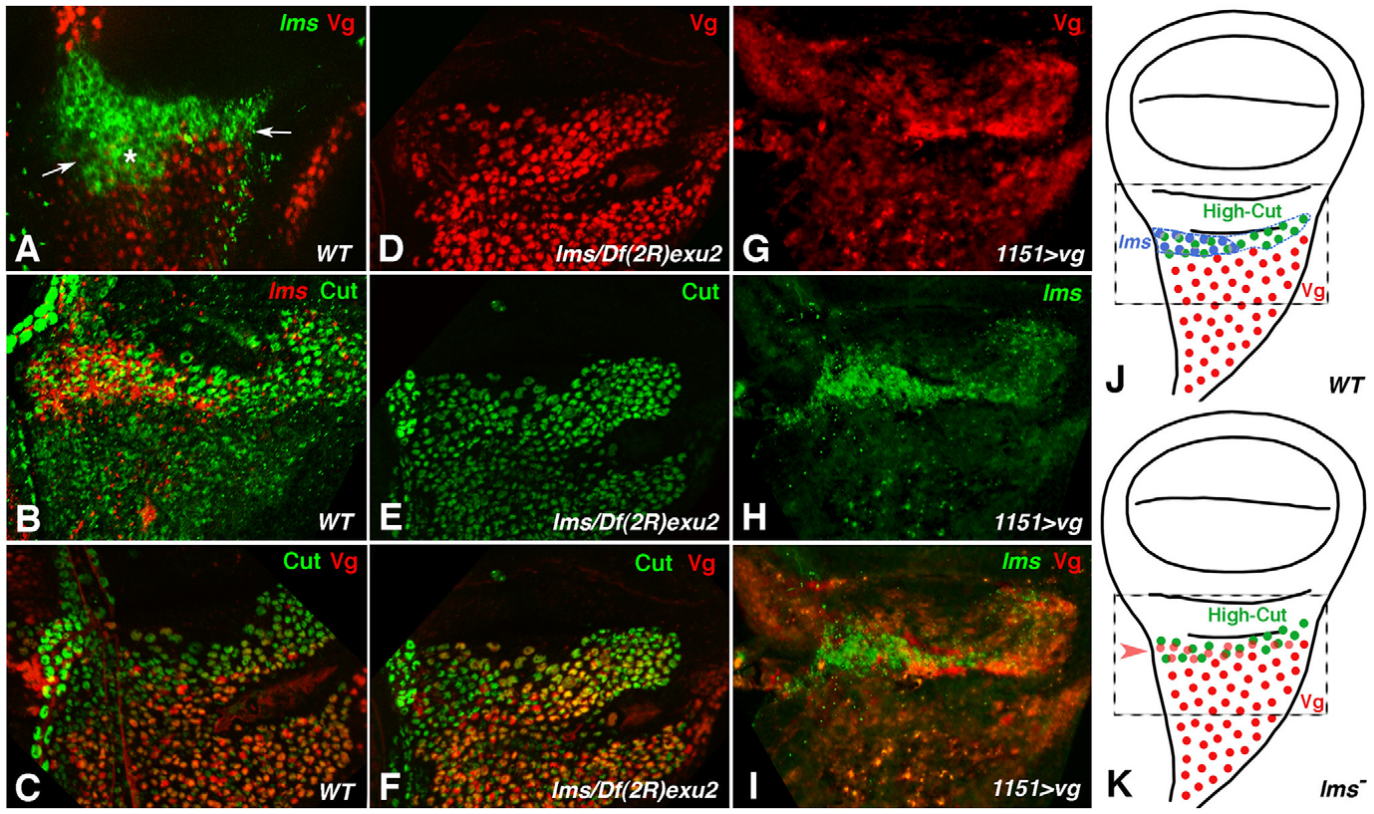
Regulatory interactions between *lms* and *vg* during patterning of adepithelial cells in wing discs. Shown are high magnification views centering on the wing hinge areas of 3rd instar wing discs (distal is up, proximal is down; anterior to the left; areas shown correspond to dashed rectangles in J, K). A-C: wild type; D-F: *lms^S95^*/*Df(2R)exu2*; G-I: *1151-GAL4*>*vg*). (**A**) *lms* mRNA expression (green) occurs in areas distally adjacent to the areas of Vg expression (red) in the adepithelial cell layer (arrows indicate border between the two domains), although there is also a small region of overlap (asterisk). (**B**) *lms* expression within the area displaying high levels of Cut protein (“high Cut domain”), which forms direct flight muscles. (**C**) Normal expression of Vg in presumptive indirect flight muscle myoblasts and high-level Cut expression in adjacent direct flight muscle myoblasts, respectively. (**D**, **E**, **F**) In *lms* mutant wing discs, Vg expression is expanded into the Cut domain. (**G**, **H**, **I**) *lms* mRNA expression in a largely normal pattern in wing disc with ectopic *vg* expression in all adepithelial cells. (**J**, **K**) Schematic drawings of Vg and Cut expression in wildtype and *lms* mutant disc, respectively, illustrating the expansion of Cut expression into anterior portions of the Vg domain upon loss of *lms* activity (area shown in panels A – I is indicated by dashed rectangle; blue dots in J represents high-level *lms* expression and region outlined with blue dotted line low-level *lms* expression area).

The restricted expression pattern of *lms* in wing discs is interesting in light of previous reports that the distal adepithelial myoblasts giving rise to direct flight muscles are marked by high levels of expression of the homeobox gene *cut,* whereas the remaining myoblasts giving rise to the indirect flight muscles are marked by the expression of *vg* and low levels of *cut* during late third instar [30]. Therefore we performed additional experiments to compare the spatial expression and clarify the regulatory relationships among *cut*, *vg*, and *lms*. In wild type wing discs, most of the *lms* expression occurs in distal adepithelial cells that are adjacent to the Vg-expressing cells (Fig. 7A, arrow), except for a small area of overlap (Fig. 7A, asterisk). Accordingly, *lms* shows extensive co-expression with high-level Cut in the anterior ∼1/2 of the distal domain, albeit with a slight extension into the low-Cut domain (Fig. 7B). Together with the published data, these observations confirm that *lms* expression is largely restricted to a subset of the adepithelial myoblasts giving rise to direct flight muscles (Fig. 7J).

**Figure 8:**
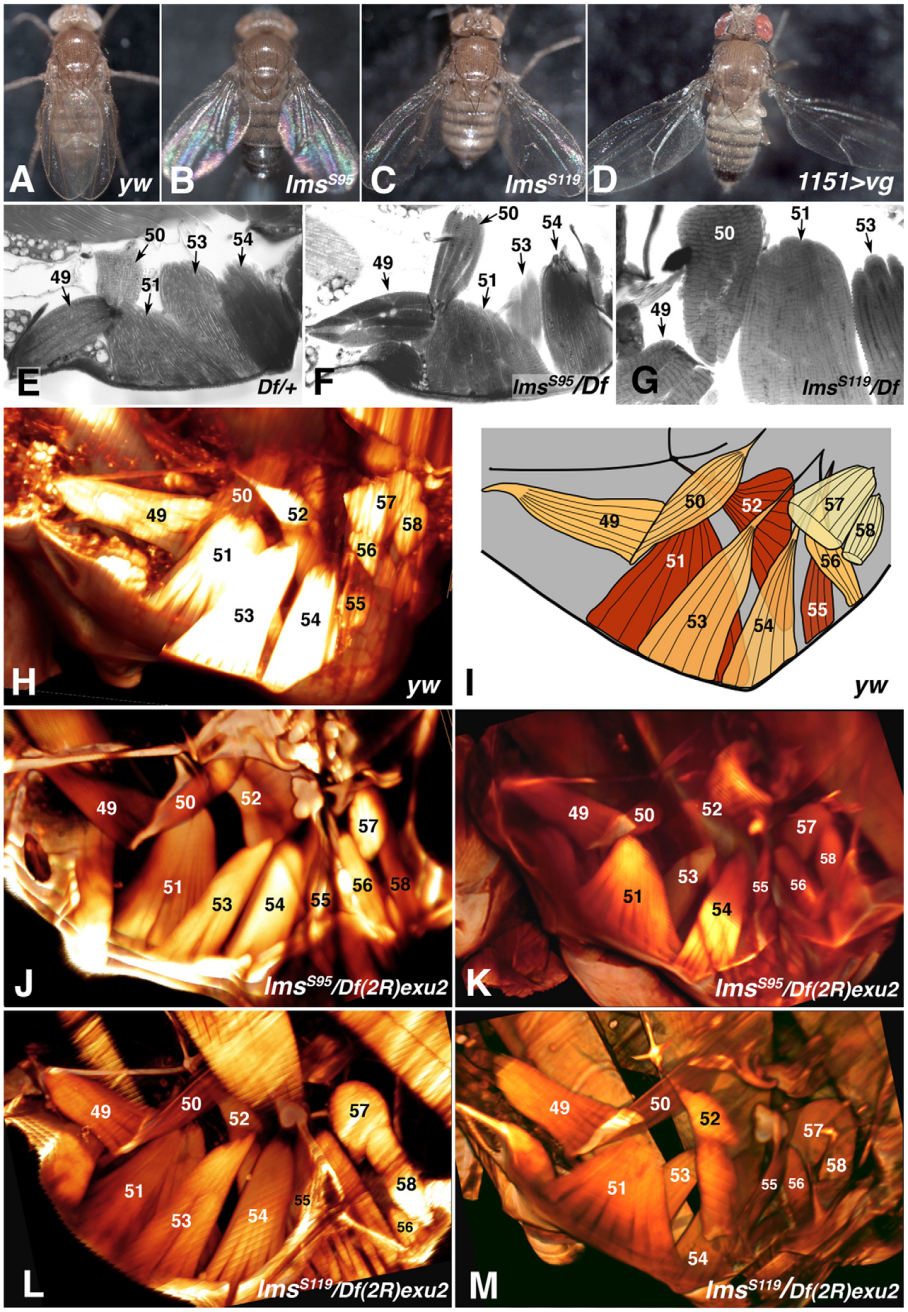
Wing posture phenotype and morphological analysis of direct flight musculature in adult *lms* mutant flies. (**A**) Control fly (*yw*). (**B**) *lms^S95^* homozygous mutant fly. (**C**) Homozygous *lms^S119^* fly. (**D**) *1151-Gal4*; *UAS-vg* fly with ectopic expression of *vg* in DFM myoblasts. (**E**) Plastic section showing DFMs from control fly (*Df(2R)exu2/+*). (**F**) Section showing DFMs from *lms^S95^/Df(2R)exu2* fly. (**G**) Section from *lms^S119^/Df(2R)exu2* fly at higher magnification showing the striated, non-fibrillar DFMs 49, 50, 51, 53 that appear normal. (**H**) and (**J** – **M**) show 3D-reconstructions obtained from stacks of images acquired by ultramicroscopy from whole mount flies. In some cases, certain areas from individual layers that covered important muscles underneath have been omitted for better clarity (see movies S2, S3, S4 with the complete reconstruction). (**H**) Control fly (*yw*) scanned from the inside. The muscles are numbered according to Miller, 1950 [46]. (**I**) Schematic drawing of direct flight muscle pattern as seen in (H) (maroon: outer muscle layer; orange: intermediate layer; yellow: inner layer). Black lines demark external cuticle and sclerites. (**J**) *lms^S95^*/*Df(2R)exu2* mutant fly scanned from the inside. (**K**) *lms^S95^*/*Df(2R)exu2* mutant fly scanned from the outside. (**L**) *lms^S119^*/*Df(2R)exu2* mutant fly scanned from the inside. (**M**) *lms^S119^*/*Df(2R)exu2* mutant (same fly as in G) scanned from the outside.

To test whether the expression of *cut* and *vg* is regulated by *lms*, we stained wing discs from *lms* mutant third instar larvae for Cut and Vg proteins. As shown in Fig. 7D-F, Vg expression is strongly expanded into the high-Cut domain in these discs such that almost all nuclei with high-Cut also contain Vg. Hence, *lms* contributes to the negative regulation of *vg* in the myoblasts of the presumptive direct flight muscles and high-Cut, which is unaltered in *lms* mutants, does not appear to be sufficient to repress *vg* (Fig. 7K). Next, we tested whether *vg* has any regulatory effects on *lms* expression. For this purpose, we forced expression of *vg* in all adepithelial cells with the *1151-Gal4* driver [25], [41]. Although we do observe a wing posture phenotype under these conditions (see also Fig. 8D), we do not detect any obvious repression of *lms* expression with ectopic *vg* (Fig. 7G-I). Thus it appears that *vg* is not sufficient to exert repressive effects on *lms* that could have fully explained the largely complementary expression of the two genes in the wing discs.

*lms* mutant flies frequently show a held-out wing phenotype, in which the wings are held at various angles (typically ∼45°) from the body axis instead of parallel to it (Fig. 8B, C, compare to A). The phenotype is seen both in flies that are homozygous for the *lms* null alleles and in flies that carry an *lms* mutation *in trans* to larger deficiencies at the locus, although the penetrance and expressivity can vary presumably due to genetic background effects (e.g., *lms^S119^/lms^S119^* or *lms^S119^/Df(2R)BSC400* escaper flies raised at 18^o^C: ∼80% penetrance of wing posture defects; *lms^S119^/Df(2R)exu2*: ∼40% penetrance). The same held-out phenotype is also seen upon RNAi knockdown of *lms* in adepithelial cells (*1151-GAL4*>*lms-IR*; data not shown). The flies with normal wing postures or with mildly held-out wings from the above-described genotypes are able to fly, but most individuals with more strongly held-out wings show poor flying capabilities or are unable to fly. In a flying assay with a 500 ml graduated cylinder according to Benzer [42], ∼45% of *lms^S119^*/*Df(2R)exu2* flies with held-out wings (n = 66) landed on the bottom, whereas for the *lms^S119^*/*Df(2R)exu2* flies with normal wing postures (n = 77) and *yw* control flies (n = 168) this number was only ∼8 and 18%, respectively (data not shown). Likewise, when *lms^S119^/lms^S119^* and *lms^S95^/Df(2R)BSC400* escaper flies with held-out wings were dropped from a height of 100 cm, all of them landed within an area of ∼25 cm diameter. When these flies were kept in an open dish or on a tip, they walked and jumped when touched, but did not fly away (e.g., see Movie S1).

The observed wing posture phenotype is similar to that of flies with ectopic *vg* expression in adepithelial myoblasts, albeit somewhat milder (Fig. 8D). The effects of ectopic *vg* have been connected to disruptions of direct flight muscles [30]. Because of the similarity of these wing phenotypes, the known role of direct flight muscles in controlling the wing posture [43], and the observed expression of *lms* within the domain of the myoblasts that give rise to direct flight muscles, we surmised that *lms* has a specific function in regulating DFM development.

Initially, we examined the direct flight musculature in dissected flies and plastic sections, which did not reveal any obvious defects in the pattern and ultrastructure (Fig. 8E – G). Therefore, we decided to analyze the DFMs in fixed whole mount thoraces via ultramicroscopy and 3D reconstruction of the scanned images, which produces detailed views of the musculature and other internal structures of the fly [44], [45]. As shown in Fig. 8H, I in a view from the inside of the thorax towards the wing attachment (see Movies S2, S3, S4), this technique has allowed us to reconstruct the morphology of *Drosophila* DFMs with great detail comparable to scanning electron micrographs [46], [47], [48] while providing the additional advantage of 3D views. Next, we used this method to examine the direct flight musculatures in *lms* mutant flies that featured strong held-out-wing phenotypes. Figures 8J and L show 3D reconstructions of the direct flight muscles from *lms^S95^*/*Df(2R)exu2* and *lms^S119^/Df(2R)exu2* flies, respectively, viewed from the inside like the control in Fig. 8H, and panels K and J depict these muscles from the same flies as viewed from the outside. Importantly, all muscles are present with their characteristic shapes, arrangements, and connections to the proper attachment sites in these mutant flies. Although there are minor differences in muscle size (e.g., compare reduced thickness of muscles 53 from the mutants in Fig. 8J with the corresponding muscle 53 from control in Fig. 8H), given the low sample size of flies that can be processed with this method (see Materials and Methods) it is unclear whether these subtle differences are related to the absence of *lms* function.

## Discussion

The homeobox gene *lms* is the first representative among its orthologs in insects and primitive chordates (*Ciona* and amphioxus) that has now been characterized in terms of its expression and function. Although some expression data are available for *Nkx-C*, its ortholog from *Ciona intestinalis*, the exact tissues of expression of this gene remain to be characterized (http://ghost.zool.kyoto-u.ac.jp/cgi-bin3/photoget2.cgi?citb089b03; [49]). Of note, in *Drosophila* the expression of *lms* is highly restricted and only found in specific domains of cells within the somatic mesoderm and the muscles derived from them. The *lms* gene is active within the somatic mesoderm during both larval and adult myogenesis, which suggested that it functions during both of these phases of muscle development.

In the embryo, *lms* is expressed like a typical muscle identity gene. Its expression in progenitors, founders and syncytia of the lateral muscles LT1 – LT4 is very similar to the mesodermal expression of the LIM homeobox gene *apterous* (*ap*), except that *ap* is activated slightly earlier in the corresponding myogenic preclusters. We have shown that *ap* exerts regulatory inputs towards *lms*, which become most apparent upon ectopic expression of *ap*. However in *ap* mutants, *lms* is still expressed largely normally and the same is true for the expression of *ap* in *lms* mutants. Thus, although regulatory interactions between the two genes do exist, their expression seems to be established largely independently from one another by related upstream activators. Two candidates for these include *msh* and *lb*. *msh* expression is significantly broader but overlaps with *lms*, and loss of *msh* function causes delayed and less robust *lms* expression. Conversely, *lb* is expressed in adjacent cells and appears to play a role in the spatial restriction of *lms* expression. Because single mutations for any of the tested candidate genes do not lead to a total disruption of *lms* expression, either these regulators act redundantly or there are additional yet unidentified regulators of *lms* expression that play more indispensable roles.

Loss of *lms* can cause the absence of individual LT muscles or in some cases morphological changes, particularly insertions into inappropriate attachment sites. The absence of an LT muscle could be due to a transformation of its identity into another, although we have not observed any clear examples of that. Alternatively, loss of *lms* function could lead to a failure of a muscle founder to acquire any specific identity or to progress only partially towards acquiring a normal LT muscle identity. We favor this second interpretation, which is compatible with the occasional presence of a small, amorphous syncytium at the position of a missing fiber and the observation of mis-attached and mis-shapen LT muscle fibers. Interestingly, similar phenotypes with comparable low expressivity were also described for *ap* mutants. To explain the low expressivity, it was proposed that additional factors can partially compensate for the loss of *ap* function [5]. Because the expression of *lms* in LT muscles and their progenitors is very similar to that of *ap*, Lms was a very good candidate for such a factor. However, in *ap*, *lms* double mutants the majority of LT muscles are still present as well, thus ruling out that the two genes are required for LT muscle specification in a mutually-redundant fashion. Rather, the roughly additive effects on the expressivity in the double mutants indicate that the two genes act in parallel with each other and in combination with yet other, perhaps more critical genes during the specification of LT muscle identities. These likely include *Kr* and *msh*, functional loss of which leads to a loss of over 30% and 50% of the LT muscles, respectively [6], [13], as well as yet unknown genes.

Altogether, it appears that *lms* (and *ap*) is needed for securing the robustness of the program determining LT muscle identities. Our findings reinforce the view that there is a significant degree of redundancy built into the muscle specification program in *Drosophila*. It is increasingly clear that the expressivities of phenotypes upon loss of function of different muscle identity genes occupy a wide range. Whereas *lms* and *ap* fall into the low end of this range, the expressivity of *msh* and *Kr* phenotypes is low for some muscle lineages and intermediate for others [13], [19]. At the high end of this spectrum are mutations for *slou*, *col*, and *eve*, which affect essentially all muscle lineages in which these identity genes are expressed [8], [9], [11]. In addition, it must be considered that identity genes act within a hierarchically structured network of interactions and at different steps of muscle development. Some of them (e.g., *slou*, *eve*, *lb*) appear to have major roles during the initial diversification of founder cells, whereas others (e.g. *ap, lms*) may act mainly or purely in the execution of identity programs of specified muscle precursors and the acquisition of individual muscle features such as shape, attachment, and distinct functional properties.

The presence of a wing posture phenotype in almost all *lms* mutant flies, albeit with variable severity, argues for a rather strict requirement for *lms* during adult muscle differentiation. The major domain of expression during this phase occupies the area of wing disc-associated myoblasts marked by high-*cut* expression that give rise to the direct flight muscles (DFMs). Interestingly, *ap* is also activated in these cells, but unlike in embryos, in this case significantly later than *lms*, namely in pupal stages. Reduction of *ap* activity has been shown to severely disrupt the formation of DFMs [29]. By contrast, all DFMs are formed and are arranged normally in *lms* null mutants, which implies that *lms* is not required for DFM muscle specification and morphogenesis. Instead, we presume that *lms* is needed in these muscles to fulfill their proper functions, which include the adjustment of wing positions and steering during flight [50]. It is conceivable that *ap* acts together with *lms* in this pathway, even though *ap* has additional roles in regulating the formation or survival of DFMs. Only the *lms* mutant flies with mild or absent held-out wings phenotypes are able to fly, but we have not examined their steering behaviour, which still may be disrupted. As shown herein, loss of *lms* leads to ectopic expression of *vg* in the presumptive DFM myoblasts. This effect could in part explain the functional defects of the resulting DFMs as GAL4/UAS-driven *vg* is known to interfere with normal DFM development [30]. In our hands, *1151-GAL4*-driven *vg* caused the reported held-out wing phenotype but, as with *lms* mutants, analysis of these flies via ultramicroscopy did not reveal any differences in the DFM muscle pattern (data not shown). Whereas most genes with held-out or held-up wing phenotypes encode various muscle proteins [9], [51], a few others such as *Dichaete* and *mirror* are expressed in proximal areas of the disc epithelium and, when mutated, cause disruptions of wing structures in the hinge region [52], [53]. By contrast, *lms* mutants with held-out wings show normal morphologies of proximal wing elements (data not shown), which together with the myoblast-specific expression pattern of *lms* reinforces the notion of a DFM-specific role of *lms*.

A similar held-out wing phenotype as for *lms* and ectopic *vg* was observed for *Wnt2* mutant flies, which show a loss, mis-attachment, or ectopic location of usually several of the DFMs in each fly [47]. Presumably as a result of these DFM patterning or attachment phenotypes, *Wnt2* mutant flies hold out their wings more strongly as compared to *lms* mutants and are also unable to fly. The late expression at the epidermal wing hinge of Wnt2 and its temporal requirement, which occurs only during pupariation, rules out a role of Wnt2 in inducing *lms*, which is already expressed during third instar. However, it remains to be examined whether Wnt2 is needed for the maintenance of *lms* expression (and/or induction of *ap*) in the developing DFM myoblasts, which would be analogous to the known role of Wingless in the maintenance of *vg* in the presumptive indirect flight muscle (IFM) myoblasts [30].

## Materials and Methods

### Drosophila stocks and genetics

The *GE11010* P-element insertion line, which contains an insertion 11nt downstream of the predicted start codon of the *lms* open reading frame (or 5′ to it if the first ATG of the longest cDNA available, EST RE33150, is being used), was purchased from Genexel (Korea) through a CNRS license. *Df(2R)exu2* and *Df(2R)BSC400* containing deletions uncovering the *lms* locus was obtained from the Bloomington Stock Center, as were the lines used for the excision screen. *Df(3R)3/1* (deleting *bap*, *lbl*, *lbe* and *C15*) was used as a *ladybird* deficiency [54]. *apME680-GFP* and *ap^UGO35^* were a gift form J. Botas (Baylor, Houston, TX [35]). *1151-Gal4* driving expression in the adepithelial cells during larval development was a gift from K. VijayRaghavan (National Centre for Biological Sciences, Bangalore) [25], [41]. *Him-GFP* was a gift from J. W. Posakony (Univ. California, San Diego) [37], [40]. *UAS-vg* was a gift from H. Skaer (Cambridge University, UK) [30]. *UAS-msh, msh^Δ68^* mutants, *msh-lacZ*, and r*P298-lacZ* were obtained from A. Nose (Tokyo University of Pharmacy and Life Sciences, Japan) [13], [55]. *UAS-ap* was a gift from J. Thomas (Salk Institute, La Jolla, CA) [5]. *UAS-Kr* was kindly provided by the Jäckle lab (MPI f. biophys. Chemie, Göttingen) [6]. Pan-mesodermal expression of muscle-identity genes were achieved with the *24B-Gal4* driver line [36] and the embryos were left to develop at 28°C for 12 hours before fixation. *lacZ-* or *GFP*-marked balancers were used throughout for the identification of homozygous mutant embryos.

For the generation of *lms^S95^ ap^UGO35^* double mutants, 50 female flies transheterozygous for the two mutations were crossed to *Sco/SM6*. To identify recombined chromosomes the lines derived from individual balanced offspring were tested for lethality in trans to *ap^UGO35^* and via PCR for the *lms* deletion.

### Generation of lms deletions

For P-excision mutagenesis, males from the *GE11015* homozygous strain were crossed with virgins from a strain containing the *Δ2*–*3* transposase on a *CyO* chromosome. Males carrying the P-element insertion together with the transposase were crossed with *w; Sco/CyO*. White eyed male progeny (either carrying *Sco* or *CyO*) were crossed individually with *w; Df(2R)exu2/SM6, eve-lacZ* females. For each of these single crosses, the lethality was scored and a balanced *GE11015-*excision/*SM6, eve-lacZ* or, if viable homozygous, stock was established. Breakpoints were determined molecularly by genomic PCR and sequencing.

### Embryo fixation

Embryos were collected on agar in Petri dishes, dechorionated for 2.5 min in 100% bleach (DanKlorix, Colgate-Palmolive), washed with H_2_O for 2 min and fixed by shaking for 20–30 min in 800 µl 5X Buffer B (50 mM K-Phosphate pH6.8, 225 mM KCl, 75 mM NaCl, 65 mM MgCl_2_), 800 µl formaldehyde, 2.5 ml H_2_O and 8 ml heptane. After removing the vitelline membrane by shaking the embryos 30 sec in methanol and two washes in methanol embryos were stored at 4°C until staining.

### In situ hybridization

4 µg of the RE33150 EST clone of *lms*, obtained from the Riken embryo library, were digested with EcoRI, gel purified with the QIAquick Gel Extraction Kit (Qiagen) and resuspended in 30 µl of DEPC-H_2_O. 10 µl of this preparation were used for a 2 h transcription reaction at 37°C using 3 µl of T3 RNA Polymerase in 30 µl final volume. 0.5 µl of the reaction was tested on a gel and the remaining probe was treated with 10 µl of 5X Carbonate Buffer (300 mM Na_2_CO_3_, 200 mM NaHCO_3_, pH 10.2) and 20 µl DEPC-H_2_O for 20 min at 65–70°C. 50 µl of 2X Stop Solution (0.2 M NaAc, pH 6.0) were added. The probe was precipitated after addition of 15 µl of 4 M LiCl, 2 µl of tRNA (50 mg/ml) and 300 µl of 100% EtOH, washed with 500 µl of 70% EtOH in DEPC-H_2_O, dissolved in 70 µl of Hybridization Solution (50% formamide, 5X SSC, 50 µg/ml heparin, 100 µg/ml tRNA, 100 µg/ml ssDNA, 0.1% Tween, pH 6.5) and used at 1∶2000 dilution for *in situ* hybridization.

### Immunohistochemistry

Whole mount embryo *in situ* hybridization, double fluorescent *in situ* hybridization and antibody labeling were performed as described in [9] and detected with Fast Red reagent (Sigma-Aldrich) or tyramide reagents (PerkinElmer). Immuno-stainings of larval imaginal discs were performed as described in [56]. *In situ* hybridization on larval discs was performed according to [57]. The stainings were either scanned with a Leica confocal microscope or recorded by using a Zeiss Apotome microscope. Ultramicroscopic images of adult flies were generated according to [44], [45]. Images were assembled using Photoshop.

The following primary antibodies were used: rabbit anti-βgalactosidase (Cappel/MP Biomedicals, CA); rabbit anti-GFP (Molecular Probes, OR); rat anti-Tropomyosin (Babraham, UK), mouse anti-Cut (Developmental Studies Hybridoma Bank, DSHB), rabbit anti-Vg (gift from S Carroll; HHMI/Univ. of Wisconsin, Madison, WI), rabbit anti-myosin (D. Kiehart, Duke Univ., NC), anti-β3-Tubulin (R. Renkawitz-Pohl, Univ. Marburg), mouse anti-Lbe ([58]), rabbit anti-Kr (1∶2000; gift from P. Carrera and G. Vorbrüggen, MPI Göttingen). For fluorescent detection, FITC-, Cy3-, or Cy5-conjugated secondary antibodies were used. Secondary antibodies were obtained from Jackson Laboratories.

### Ultramicroscopy

Ultramicroscopy (UM) is a microscopy technique allowing three-dimensional reconstructions of up to cm-sized specimen with micrometer resolution using a laser light sheet [44], [45]. Using this technique, we performed three-dimensional reconstructions of the inner anatomy of chemically cleared entire *Drosophila* flies [59]. Flies were anaesthetised by ether, fixed in 4% paraformaldehyde overnight, and dehydrated in an ascending ethanol series (50%, 70%, 96%, 2×100% for 6 h). Afterwards, they were incubated in a clearing solution consisting of 2 parts benzyl benzoate and 1 part benzylalcohol (Sigma-Aldrich, Germany) for 3 days. Imaging the flies' inner morphology was performed using autofluorescence of *Drosophila* muscles. Autofluorescence was excited at 488 nm laser light sheet having a beam diameter of approx. 3.2 µm at the focus. Images were recorded with a 10X objective (NA 0.30) using a CCD camera (CoolSnap K4, Roper Scientific) as described in [44]. Two individuals from each genotype were analyzed (*yw, lms^S95^*/*Df(2R)exu2*, *lms^S119^*/*Df(2R)exu2*) and the mutant flies utilized showed strong held-out wing phenotypes.

## Supporting Information

Movie S1 lms[S119] homozygous flies. Non-flying behaviour of homozygous lmsS119 flies in open dish.
(1.90 MB MOV)

Movie S2 yw control inside. Scan of yw control fly from the inside (anterior to the right) (see also Fig. 8H).
(0.80 MB MP4)

Movie S3 lms[S95]/Df(2R)exu2 inside. Scan of lmsS95/Df(2R)exu2 fly from the inside (anterior to the right) (see also Fig. 8J).
(0.95 MB MP4)

Movie S4 lms[S119]/Df(2R)exu2 outside. Scan of lmsS119/Df(2R)exu2 fly from the outside (anterior to the left) (see also Fig. 8M).
(0.98 MB MP4)
